# Scrotal Paget disease with epidermolytic acanthoma

**DOI:** 10.1016/j.jdcr.2026.03.044

**Published:** 2026-03-27

**Authors:** Jia-Feng Li, Zhou-Hong Tan, Yu-Chen Zhuang, Yu-Zhe Sun

**Affiliations:** a Dermatology Hospital, Southern Medical University, Guangzhou City, Guangdong Province, China; b The Skin Department, Hechi Third People’s Hospital, Hechi City, Guangxi Province, China

**Keywords:** epidermolytic acanthoma, epidermolytic hyperkeratosis, extra-mammary Paget disease, pathology

A 55-year-old male patient presented to our clinic with complaints of red-brown papules on the penis and scrotum, which had appeared over the past 2 years. The lesions were associated with itching and pain, gradually expanding in size. Recurrent scratching resulted in the coalescence of these lesions into hypertrophic plaques with lichenoid changes and pigmentation at the edges. The patient had previously been diagnosed with eczema at another dermatology clinic and treated with topical steroids (exact medication not specified), but without improvement. He denied fever, dysuria, or any other systemic discomfort, and had no significant medical history.

On examination, large erythematous plaques (approximately 5.5 × 4.5 cm) were noted on the penis and scrotum, surrounded by small skin-colored papules, with scattered pigmentation, localized lichenoid changes, and no ulceration (Fig 1, *A*). Bilateral inguinal lymph nodes were not enlarged. Preoperative contrast-enhanced Computed Tomography scans of the chest, abdomen, and pelvis revealed a possible image feature for pancreatic neuroendocrine tumor, and no other definite evidence for malignancy was found. Based on clinical findings, Paget disease was clinically diagnosed, and Mohs micrographic surgery was performed to remove the lesion.

**Fig 1:**
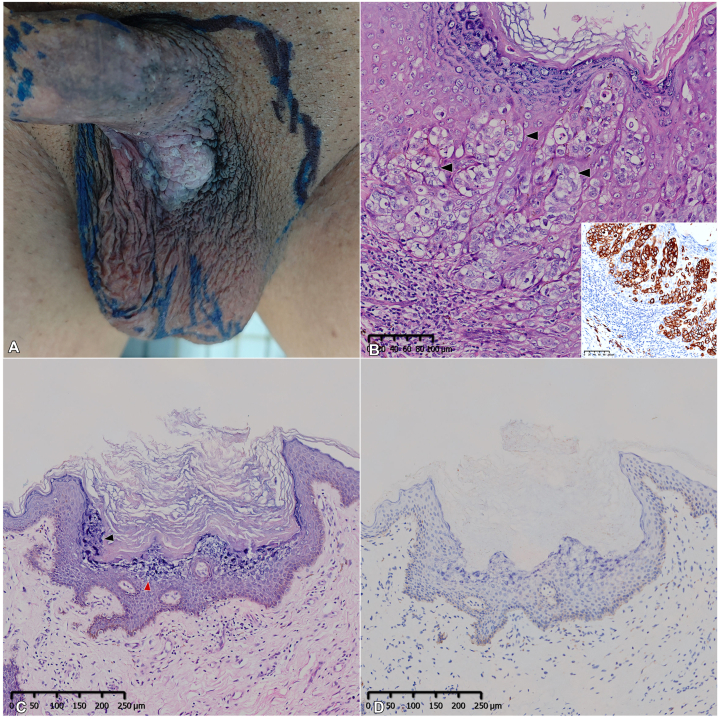
**(A)** Clinical presentation showing erythematous plaques on the penis and scrotum. **(B)** Histopathologic examination showing tumor cells (indicated with *arrowheads*) scattered in the epidermis. Immunohistochemical staining for CK7 shown in the bottom right inset. **(C)** Histopathologic features of funnel-shaped epidermal hyperplasia, excessive keratinization, granular layer degeneration (indicated with *black arrowhead*), and vacuolated cells caused by granular layer degeneration (indicated with *red arrowhead*) found at the Mohs surgical margin. **(D)** Immunohistochemical staining for CK7 was negative at the EA area of the surgical margin. *EA*, Epidermolytic acanthoma; *CK7*, Cytokeratin 7.

Postoperative histopathologic examination revealed irregular epidermal hyperplasia with numerous tumor cells (Paget cells) scattered within the epidermis (Fig 1, *B*, tumor cells indicated with arrowheads). The tumor cells varied in size, with abundant cytoplasm and readily visible mitotic figures, which showed strong positivity for Cytokeratin 7 (CK7) by immunohistochemical staining (the small inset at the bottom right corner of Fig 1, *B*). At the Mohs surgical margins (Fig 1, *C*), several areas of funnel-like squamous epithelium proliferation with excessive keratinization, granular layer degeneration and acantholysis were observed (indicated with arrowheads). Immunohistochemical staining for CK7 was negative in the margin tissue (Fig 1, *D*), demonstrating that there were no Paget cells in the surgical margin tissues. Regarding the possibility of pancreatic neuroendocrine tumor suggested by imaging findings, we conducted an extensive literature review. Currently, there is no evidence to support an association between pancreatic neuroendocrine tumors and Paget disease of the skin. Therefore, based on both clinical and histopathologic findings, the diagnosis of primary scrotal Paget disease with epidermolytic acanthoma (EA) was eventually made.

EA usually manifests as white to flesh-colored papules, often less than 1 cm in diameter, predominantly affecting the scrotum, head, neck, and lower limbs, which can present in either solitary form or disseminated form.^1^ EA is considered to result from genetic mutations, trauma, enhanced keratinocyte metabolic activity,^2^ and possibly Human Papillomavirus infection.^3^ Clinically, EA is often mistaken for condyloma, common warts, or seborrheic keratosis.^4^ Therefore, histopathological examination is crucial for accurate diagnosis. The key histologic feature of EA is epidermolytic hyperkeratosis (EHK), which presents with vacuolization of the granular and upper spinous layers (roughly 50% or more of the surface of the lesion),^4^ irregular-shaped keratin granules within cells, and eosinophilic inclusions in the spinous cells. Another vital histological feature of EA is epidermal hyperplasia, which could present as papillomatous, acanthotic, or funnel-shaped.^1^ Notedly, although EHK is characteristic of EA, it is not always specific and can also occur in conditions like epidermolytic ichthyosis or epidermolytic epidermal nevi.^4^

Extramammary Paget disease, first described by Crocker in 1889, is a low-grade malignancy that typically affects areas rich in apocrine glands, such as the vulva, axilla, groin, scrotum, and perianal region.^5^^,^^6^ It commonly presents as itchy erythematous patches, gradually progressing into painful plaques with thickened, rough, and well-demarcated areas that can have eczematous features such as erosions, exudation and crusting.^7^ Histologically, such lesion appearances are associated with Paget cells presenting in the epidermis, with nest-like patterns or gland-like structures, which are malignant glandular epithelial cells with abundant clear cytoplasm (rich in mucin) as well as pleomorphic and hyperchromatic nuclei.^6^

Anil et al^8^ have reported a case of extramammary Paget disease associated with EHK, which shares some similarities with our case. However, in their study, the EHK region of the patient's skin lesion exhibited positive CK7 staining, leading the authors to conclude that the lesion histologically represented EHK-like Paget cells, rather than Paget cells co-existed with EHK. In contrast, we revealed that the EHK region at the surgical margin of our case did not contain Paget cells (CK7-negative) and exhibited histologic features consistent with EA. This suggests that the patient had both extramammary Paget disease and EA, which is an exceptionally rare co-occurrence. We hypothesize that the chronic pruritus and recurrent scratching associated with Paget disease may have contributed to the development of EA.^9^ Notably, the vacuolated cells seen in EA can easily be misinterpreted as Paget cells, which may lead to a misdiagnosis of positive surgical margins and result in unnecessary extensive excisions. Moreover, differential diagnoses should also include conditions such as scrotal eczema, Hailey–Hailey disease, and pemphigus vegetans, where careful histopathologic evaluation and immunohistochemical staining can provide crucial diagnostic insights. In terms of treatment, after undergoing Mohs micrographic surgery, the patient had a favorable postoperative outcome, with no recurrence at 16 m of follow-up. This suggests that the coexistence of EA does not alter the therapeutic approach for scrotal Paget disease.

## Conflicts of interest

None disclosed.
